# The complete chloroplast genome of *Nicotiana plumbaginifolia*

**DOI:** 10.1080/23802359.2021.2024772

**Published:** 2022-01-24

**Authors:** Zhijun Tong, Tianyu Du, Yiyu Hu, Haijun Chen, Enhui Shen, Longjiang Fan, Bingguang Xiao

**Affiliations:** a Key Laboratory of Tobacco Biotechnological Breeding, Yunnan Academy of Tobacco Agricultural Sciences, Kunming, China; b College of Agriculture and Biotechnology, Institute of Crop Sciences, Zhejiang University, Hangzhou, China; c Center for Bioinformatics and Big Data Technology, The Rural Development Academy, Zhejiang University, Hangzhou, China

**Keywords:** Chloroplast genome, tobacco, *Nicotiana plumbaginifolia*

## Abstract

*Nicotiana plumbaginifolia* Vivianiis 1802 is an annual herb, native to Mexico and South America. It is one of the most widely distributed tobacco species. As a wild tobacco, *N. plumbaginifolia* has provided several economically important disease-resistance genes to cultivated tobacco. We assembled the complete chloroplast genome of *N. plumbaginifolia*. The chloroplast genome is 155,945 bp in length, which includes a large single copy region (86,621 bp), a small single copy region (18,528 bp) and two separated inverted repeat regions (25,398 bp). A total of 117 unique genes were annotated, consisting of 84 protein-coding genes, 29 tRNA genes and 4 rRNA genes. Based on chloroplast genomes of 17 *Nicotiana* species, phylogenetic analyses indicated that *N. plumbaginifolia* was closely related to *N. suaveolens* and *N. amplexicaulis*.

Species of the genus *Nicotiana*, including herbaceous plants and shrubs in the family Solanaceae, are used for smoking, ceremonial, or ornamental purposes (Lewis 2020). *Nicotiana plumbaginifolia* is commonly called Tex-Mex tobacco and wild tobacco and exhibits antimicrobial and antioxidant activities (Ajaib et al. 2016). It has been treated as an important donor of genes/beneficial alleles for tobacco breeding. A fair number of disease-resistance genes have been transferred to cultivated tobacco from *N. plumbaginifolia*, including black shank resistance and tobacco cyst nematode (*Globodera tabacum*) resistance (Johnson et al. 2009). Chloroplast genomes helps us understand the origin and evolution of plants. Previously, *Nicotiana* was divided into 13 sections based on multiple chloroplast markers (Clarkson et al. 2004). *N. plumbaginifolia* was divided into the section *Alatae*, which is considered as a monophyletic group (Kaczorowski et al. 2005). The species epithet ‘plumbaginifolia’ comes from the way in which the leaves resemble those of species in the genus *Plumbago*. Because of the complex development history of polyploidy and hybridization, *N. plumbaginifolia* and other *Nicotiana* species are also used as evolutionary model systems. Chloroplast genomes are also related with important crop traits such as yield, crop quality, resistance to disease and pest (Jin and Daniell 2015).

Here, we assembled the plastid genome of *N. plumbaginifolia.* The sample of *N. plumbaginifolia* was collected in Brazil, near Santa Catarina (27°3.462 S, 51°6.538 W) and deposited in the Herbarium of Zhejiang University (accession number: HZU60244006). Total genomic DNA was sequenced by the Illumina platform. After quality control with NGSQCToolkit v2.3 (Patel and Jain 2012), the high quality data was applied in *de novo* assembly by NOVOPlasty v3.6 (Dierckxsens et al. 2017) using the *Nicotiana tabacum* complete chloroplast genome (GenBank accession number: NC_001879) as a reference. Genome annotation was performed by the GeSeq online (Tillich et al. 2017). The assembled genome sequences and annotation information have been submitted to the DNA Data Bank of Japan under accession number LC649170.

The total length of *N. plumbaginifolia* chloroplast genome is 155,945 bp. Like most angiosperm chloroplast genomes, this genome exhibited a distinct quadripartite structure, including a pair of inverted repeats (IRa and IRb, 25,398 bp each), the large single-copy region (LSC, 86,621 bp) and the small single-copy region (SSC, 18,528 bp). The GC contents of the IR, LSC, and SSC regions are 42, 35, and 30%, respectively. A total of 117 unique genes were annotated. Among these, there are 84 protein-coding genes, 29 tRNA genes and 4 rRNA genes.

More recently, phylogeny inference based on complete chloroplast genomes provided insights into the phylogeny of certain families and genera (Amiryousefi et al. 2018). To investigate the evolutionary position of *N. plumbaginifolia* among *Nicotiana* species, we built a phylogenetic tree of 16 *Nicotiana* species based on complete chloroplast genome sequences downloaded from the NCBI GenBank database*. Solanum lycopersicum* was used as an outgroup. We first performed alignment by MAFFT v7.310 (Katoh et al. 2002) with the default parameter. Then, IQ-tree v1.6.12, an effective algorithm for estimating maximum-likelihood phylogenies, was used to construct a phylogenetic tree with recommended model TVM + F+R2 and 1000 bootstrap values (Nguyen et al. 2015). Finally, the tree was illustrated and modified using iTOL (Letunic and Bork 2019).

The phylogenic tree showed that *N. plumbaginifolia* first clustered with *N. suaveolens* and *N. amplexicaulis* forming as a monophyletic group (Figure 1). The young allotetraploid *N. tabacum* arose through the hybridization of the ancestral parents *Nicotiana sylvestris* and *N. tomentosiformis* (Edwards et al. 2017). The phylogenetic relationship between *N. tabacum and N. sylvestris* was quite close, which supported the assumption that *N. sylvestris* was the maternal genome donor (Figure 1). Our results provide basic information for further phylogenetic analysis on the genus *Nicotiana*.

**Figure 1.**
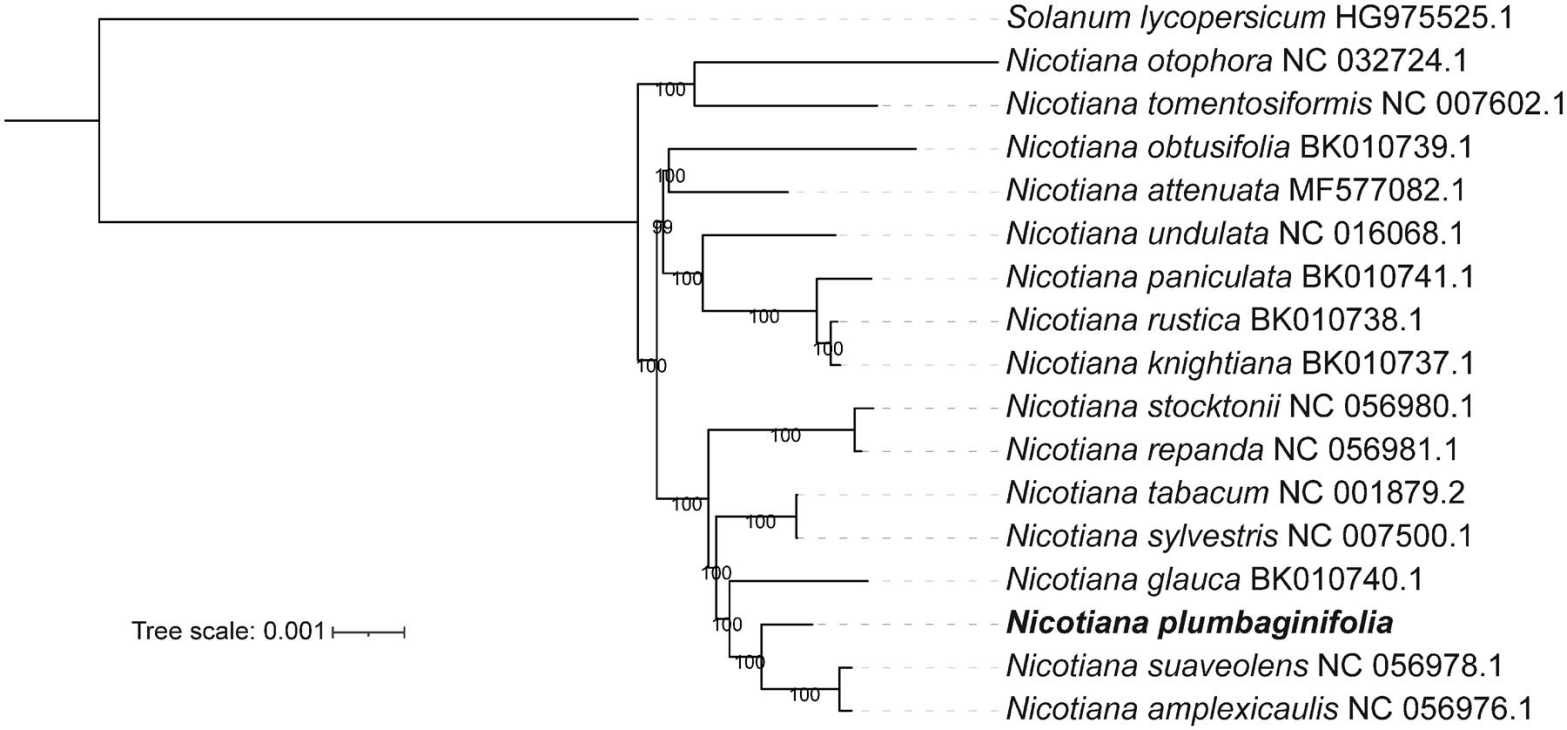
Maximum likelihood (ML) phylogenetic tree based on 16 *Nicotiana* species, using *S. lycopersicum* as an outgroup. The numbers on the node are the fast bootstrap value based on 1,000 replicates. The analyzed species and corresponding Genbank accession numbers are as follows: *N. amplexicaulis* NC_056976.1; *N. attenuate* NC_036467.1; *N. debneyi* NC_056977.1; *N. glauca* NC_056979.1; *N. otophora* NC_032724.1; *N. repanda* NC_056981.1; *N. stocktonii* NC_056980.1; *N. suaveolens* NC_056978.1; *N. sylvestris* NC_007500.1; *N. tabacum* NC_001879.2; *N. tomentosiformis* NC_007602.1; *N. undulata* NC_016068.1; *N. knightiana* BK010737; *N. rustica* BK010738; *N. paniculate* BK010741; *N. obtusifolia* BK010739; and *S. lycopersicum* HG975525.1.

## Data Availability

The data of this study is available in the DNA Data Bank of Japan (https://www.ddbj.nig.ac.jp/) under the accession number LC649170. The associated Bioproject, SRA, and Bio-Sample numbers are PRJNA763728, SAMN21448217, and SRR15927287, respectively.
